# Clinical Contribution

**Published:** 1893-04

**Authors:** T. D. Stow

**Affiliations:** Mexico, N, Y.


### CLINICAL CONTRIBUTION.

T. D. Stow, M. D., Mexico, N. Y.

### Psorinum$^{CM}$.

Arthur D——, an alumnus of Middletown College, Conn., returned to his home in Mexico, N. Y., to spend the holidays of '91. Soon after his return his father came to me and stated as follows: "Arthur is in his twenty-first year, and has been away some ten months. For the last sixty days has had a papular eruption on his hands, forearms, between the fingers, in the popliteal and elbow flexures, with much intolerable itching, bleeding, and burning after scratching. Has much thirst for cold water, taking much at a time. Itching worse at night and when warm in bed; sweats easily; feels very weak and grows thin; has much anxiety regarding his condition."

I sent him one dose of Psorinum$^{cm}$ (Swan). At the expiration of thirty-six hours there was marked amelioration of all the symptoms. At the end of three days no more itching or burning; less perspiration and less debility. In a week's time his hands, wrists, and skin were free from any eruption, and he was cured. He has remained well ever since.

Earle Mentor, a lad of eight years, had a papulo-vesicular eruption over the entire surface, but more in the flexures of joints, on hands, between fingers, on the wrists, and in the palms, where it closely resembled eczema. The itching was so intolerable that he scratched until blood flowed, after which the itching ceased and he would fall asleep. Aggravation at night on undressing, and in bed; general perspiration at night, during sleep, but giving no relief; his face looked sallow; he was pale also; tongue coated a dirty white; he had great thirst for cold water; odor from body offensive.

I first saw him in September, '91. He then also had periostitis of the right tibia just below the tuberosity, with abscess, much tumefaction, much inflammation, and temperature 101°. The right leg was studded with the eruption more thickly than any other part. The boy's temper was morose.

After opening and irrigating the abscess and limb, and carefully dressing the same, gave him two doses of Merc-sol.$^{500}$, and awaited its action. Some improvement followed, but it was not satisfactory. Carefully reviewing his case, I gave him Psorinum$^{cm}$ (Swan), three powders, a powder to be taken every twelve hours. Great aggravation of all his symptoms followed, but in the course of a week improvement commenced. Meantime it became necessary to cut down upon the tibia, drill into its cavity, and remove a small sequestrum. From that day the boy rapidly recovered without further medication.

Myrna Fellows, aged fourteen, bright and active, had herpes on his arms, wrists, hands, between fingers, on body, legs, feet. Intolerable itching, worse when warm, and at night; skin of hands cracks and bleeds; the skin inflames. Gave Psorinum$^{cm}$ (Swan), one dose. Improvement immediately followed, and in one week he was well. Has remained so up to date.

### Pneumonia-vera.

J. D. H——, a retired lawyer, some fifty-eight years old, is addicted to inebriety. Last October he had a short but sharp attack of pneumonia.

When I saw him for the first time during his attack, he had short, sharp pain in both lungs; dull, full pain in same, more in the right side, with dry, hard cough, increasing the pain; much thirst; chilliness on stirring in bed; great anxiety, with restlessness. His head ached hard in forehead.

Circumscribed flush on both cheeks—rather purple flush.

Preferred the dorsal decubitus.

Pulse full, sharp, quick, frequent, 120 to 130 per minute.

Respiration short, frequent, constricted, 30 per minute.

Gave Aconite$^{30}$ in water, repeating every hour.

Profuse perspiration followed in the course of an hour, increasing so that his linen and bed-sheets and one blanket were wet. Discontinued the Acon., and gave nothing more for one day. All his symptoms gradually disappeared, leaving but little more than a blood-streaked, free, quite abundant expectoration, with great thirst for large quantities of cold water; some pain in right side when coughing and when turning in bed, for which Bry.$^{cc}$ was given with speedy relief. He recovered rapidly. I gave him Acon. not more than four times, and he had but one prescription of Bry. I report this case as nothing more than has been seen by most any homœopathic physician, but as illustrative of the effect of homœopathic remedies, in treating pneumonia, pleurisy, etc., as compared with the slow-coach and death-dealing processes of old-school therapeutics.

Grippe and pneumonia were with us last winter in aggravated forms, and the mortality and wrecks of humanity were simply appalling.

The case also illustrates the benefits of Homœopathy among the dissipated and enfeebled. I had many cases of bronchitis and pneumonia—some of them grave—and all speedily recovered and remain well.

